# Adequacy of Surgical Pathology Reports of Colorectal Carcinoma and Its Significance

**DOI:** 10.7759/cureus.16965

**Published:** 2021-08-06

**Authors:** Salahuddin Khan, Ghulam Haider, Zain Abid, Neelma Bukhari, Shah Zeb Khan, Masooma Abid

**Affiliations:** 1 Medical Oncology, Jinnah Postgraduate Medical Centre, Karachi, PAK; 2 Clinical Oncology, Bannu Institute of Nuclear Medicine Oncology and Radiotherapy, Bannu, PAK; 3 Medicine, Jinnah Medical and Dental College, Karachi, PAK

**Keywords:** colorectal neoplasm, pathology report, standardization, prognostic factors, lymph nodes, international guidelines

## Abstract

Introduction

Colorectal cancer is the fifth most common cancer in the world. For loco-regionally confined disease surgery is the definitive treatment. An adequate surgical pathology report is mandatory for the selection of adjuvant therapy. The objective of this study is to analyze whether adequate information is provided or not in the surgical pathology reports of colorectal carcinoma as according to College of American Pathologists (CAP) guidelines.

Method

This is a cross-sectional study carried out in the Department of Clinical Oncology, Jinnah Postgraduate Medical Center (JPMC) Karachi, tertiary care hospital in Pakistan. The duration of the study was from February 2020 to January 2021. A total of 153 surgical pathology reports issued by 11 different hospital-based laboratories after definitive surgery was assessed to look at its concordance rate with the checklist adapted from the CAP guidelines.

Results

Out of 153 surgical pathology reports, clinical information was provided in 72.5% of reports. Details of tumor extension were present in 88.2%, tumor margin in 75%, surgical procedure in 79%, and tumor deposits in 39.2% of reports. Macroscopic details including tumor perforation and evaluation of mesorectum were documented in 51.6% and 53.5% of the reports respectively. Details regarding perineural invasion along with lymphovascular invasion were present in 81.6% and 93% of the reports, respectively. The treatment effect was documented in only 25% of reports and regional lymph node status has been described in 85% of reports. Parameters described in all surgical pathology reports were: tumor site, tumor type, histologic type, and histologic grade. The pathological stage of the disease was documented in 91.5% of the reports.

Conclusion

This study concluded that surgical pathology reports of the majority of pathology laboratories were not fully adhered to the checklist provided by the CAP guidelines. This will affect post-operative management along with the prediction of disease prognosis.

## Introduction

Colorectal cancer is the most common gastrointestinal malignancy. It is the third most common cause of cancer, while the fourth commonest cause of death in the world [1]. Colon cancer incidence rate is equal in males and females, but rectal cancer is slightly predominant in the male population [1,2]. The prevalence of colorectal cancer disease is high in developed countries [2]. Survival of colorectal cancer depends upon the stage of disease at diagnosis and typically ranges from a 90% 5-year survival rate for cancers detected at the localized stage; 70% for regional; to 10% for stage IV disease [3]. The probability of colorectal cancer is progressively increasing from 40 years of age and rising significantly after the age of 50 years [1,3]. More than 90% of colorectal cancer cases occur in people aged 50 or older [3]. Colorectal cancer appears to be increasing among the younger population due to adaptation of unhealthy lifestyle [4] and genetic predisposition.

In Asia, Pakistan is a low-risk country for colorectal cancer; however, recent studies have reported a rise in colorectal cancer cases in patients above the age of 50 years [5]. Over the next few decades, it is predicted that there will be a rapid rise in colorectal cancer cases due to lifestyle modification in Pakistani society. Decreased physical activity, obesity, high intake of preserved and processed food, red meat, and smoking are associated with an elevated risk of colorectal cancer.

A multidisciplinary team is required for the management of colorectal cancer, which includes radiologists, colorectal surgeons, pathologists, radiation oncologists, and medical oncologists. Surgical resection is the main curative treatment for rectal adenocarcinoma. However, surgery alone is associated with a high risk of local and distant recurrence. Therefore, multi-modality treatment with the incorporation of radiotherapy and chemotherapy followed by surgery to reduce the risk of cancer recurrence is mandatory. In colon cancer pattern of failure is predominantly distant [6], while in rectal cancer the pattern of failure is locoregional.

Adequate information in surgical pathology reports of colorectal cancer must provide important information to select patients for appropriate adjuvant therapy and to predict disease prognosis [7]. For example, adjuvant chemotherapy has proven to be beneficial if nodal involvement is documented after adequate nodal dissection. (Dukes' C cases) [8]. In rectal cancer, due to close and positive margins, there is an increased risk of local recurrence which needs adjuvant radiotherapy [9]. The most powerful predictors of postoperative outcome in colorectal cancer include pathologic stage, histologic grade, lymphovascular invasion, perineural invasion, and tumor resection margins [10].

Survival of colorectal cancer depends upon the stage of disease at diagnosis and typically ranges from a 90% five-year survival rate for cancers detected at the localized stage; 70% for regional and 10% for stage IV cancer [3]. Due to inadequate information in the surgical pathology reports of colorectal cases, patients may be either over-treated or under-treated leading to significant morbidity, mortality, and financial burden. No local guidelines have been developed until now in Pakistan for surgical pathology reporting of any cancer. To date, only one laboratory has been accredited by the CAP in Pakistan. However, the majority of laboratories included in our study were following the CAP guidelines to some extent.

The objective of this study is to analyze whether adequate information is provided in pathology reports after resection of colorectal cancer as per the recommendation of the CAP guidelines. This will help medical oncologists to decide adjuvant treatment modalities, intensity, and duration of treatment along with the prediction of disease outcome in the future.

## Materials and methods

This is a cross-sectional study in which we reviewed 153 surgical pathology reports of colorectal cancer issued by 11 different hospital-based laboratories of Karachi city. All patients were presented to the Clinical Oncology Department of JPMC, Karachi with surgical pathology reports for further oncological treatment. This study was done over a year from February 2020 to January 2021. Institutional Ethical Review Committee approval was taken before starting the study. Surgical pathology reports of patients who underwent curative surgery during the study duration were included in the study; however, those who did not undergo curative surgery were excluded from the study. In addition, specimens including simple diagnostic biopsy and trans-anal disk excision were also eliminated from the study. Adequacy of surgical pathology reports of colorectal cancer was defined as the percentage/number of reports which has documented all elements of the checklist adopted from CAP guidelines (Table 1) entitled as "Protocol for the examination of resection specimens from patients with primary carcinoma of the colon and rectum" (Version:4.1.0.0, February 2020. https://documents.cap.org/protocols/cp-gilower-colonrectum-resection-20-4100.pdf).

**Table 1:** Checklist adopted from CAP guidelines for documentation of elements in surgical pathology report of colorectal cancer. *TNM: Tumor size, Lymph Nodes, Metastasis. +Data elements preceded by this symbol are not required for accreditation purposes. CAP: College of American Pathologists.

Checklist Adopted from CAP Guidelines for Documentation of Elements in Surgical Pathology Report of Colorectal cancer
Clinical information
Procedure
Tumor type
Tumor site
Tumor perforation
Evaluation of mesorectum
Tumor extension
Margins
Lymphovascular invasion
Perineural invasion
Histologic type
Histologic grade
Tumor deposits
Treatment effect
Regional lymph node status
Pathological TNM* stage
TNM* descriptors
Presence or absence of tumor budding+
Presence or absence of polyps+

All elements (parameters) were assessed for the presence, absence, and documentation of details on each report. Data was entered in SPSS version 20 (IBM Corp., Armonk, NY). T-test and Chi-square were used for analysis. P-values less than 0.05 were considered significant.

## Results

**Figure 1:**
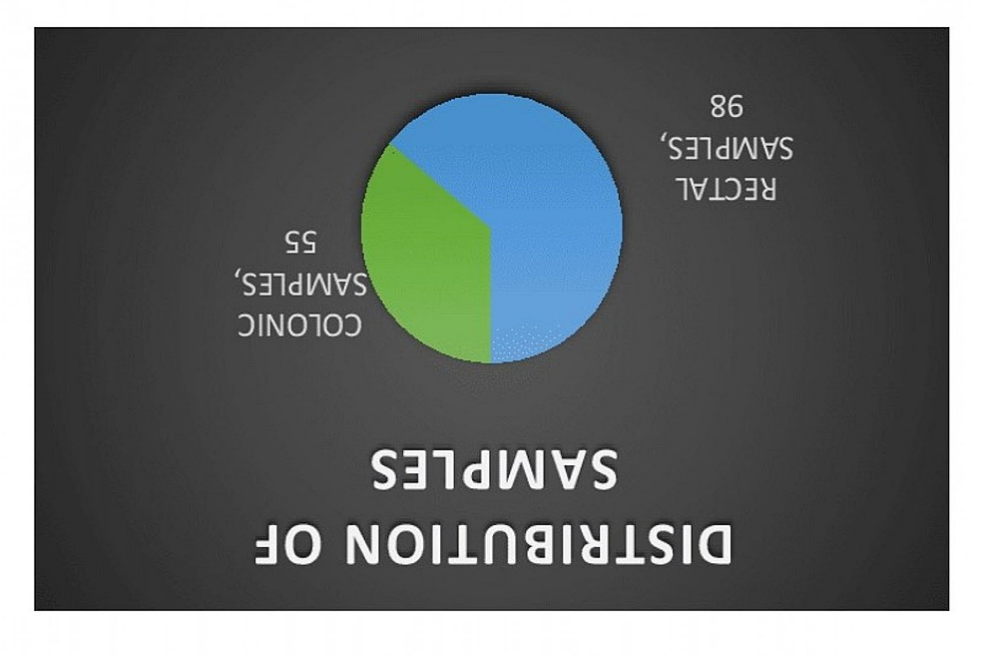
Distribution of sample.

A total of 153 surgical pathology reports of colorectal carcinoma were assessed during the study duration from different laboratories. Out of 153 patient reports, 98 (64%) were from the rectal samples, while 55 (36%) were from the colon resected specimens (Figure 1). 

We evaluated the surgical pathology reports as per the recommendation of CAP guidelines. The majority of pathology reports included in our study were associated with private hospital laboratories. None of the surgical pathology reports showed 100% concordance with the checklist adopted from the CAP guidelines (Table 1). In our study, 72% of pathology reports showed clinical information. However, 79% and 100% of reports showed procedure type and tumor type, respectively. Tumor site, histologic type, and grade of tumor were documented in all pathology reports. Details of lymphovascular invasion and perineural invasion were present in 93% and 81.6% of reports, respectively. Macroscopic details of tumor perforation and evaluation of mesorectum were present in 51.6% and 53.5% of pathology reports, respectively. In our study, only 25% of reports documented treatment effects. Tumor deposits and excision margin status were mentioned in 39.2% and 75% of the reports, respectively. The presence or absence of tumor budding was indicated in 55.5% of reports. Out of which 24.7% reports showed the presence of tumor budding. However, 51% of the reports commented about polyps and out of them, 30.7% of reports indicated the presence of polyps. Pathological staging was mentioned in 91.5% of reports, while TNM descriptors were indicated in only 35.9% of reports. Please note that 85% of reports documented the excised regional lymph nodes status (Table 2).

**Table 2:** Performance level of laboratories indicating number of reports describing each element as per CAP guidelines checklist. CAP: College of American Pathologists.

Parameters	Number of reports	Percentage
Clinical information		
Present	111	72.5%
Absent	42	27.4%
Procedure		
Documented	121	79%
Non-documented	32	20.9%
Tumor type		
Present	153	100%
Absent	0	0%
Tumor site		
Present	153	100%
Absent	0	0%
Histologic type		
Present	153	100%
Absent	0	0%
Histologic grade		
Present	153	100%
Absent	0	0%
Lymphovascular invasion		
Documented	141	93%
Non-documented	12	7.8%
Perineural invasion		
Documented	125	81.6%
Non-documented	28	18.3
Tumor deposit		
Documented	60	39.2%
Non-documented	93	60.7%
Treatment effect		
Documented	30	25%
Non-documented	123	80.3%
Resected margins		
Explained	114	75%
Not explained	39	25.4
Tumor extension		
Documented	135	88.2%
Non-documented	18	11.7%
Tumor perforation		
Documented	79	51.6%
Non-documented	74	48.3
Evaluation of mesorectum		
Documented	82	53.5%
Non-documented	71	46.4
Lymph node status		
Present	130	85%
Absent	23	15.0%
Pathological stage (pTNM)		
Present	140	91.5%
Absent	13	8.4%
TNM descriptors		
Present	55	35.9%
Absent	98	64.0%
Tumor budding		
Present	21	24.7%
Absent	64	75.2%
Non-documented	68	44.4%
Polyps		
Present	24	30.7%
Absent	54	69.2%
Non-documented	75	49.0%

## Discussion

In colorectal cancer, biological features and the extent of disease are the factors that affect the risk and prediction of treatment response. Medical oncologists assessed these features based on surgical pathology reports made by a pathologist. Many surgical pathology reports were unable to mention elements required for postoperative treatment planning. Many international guidelines had developed a protocol for surgical pathology reporting of colorectal cancer to improve its quality. In our study, we assessed that whether all-important parameters were present in the pathology reports or not. It is to analyze the adequacy of surgical pathology reporting of colorectal carcinoma of various laboratories.

In our study parameters like tumor type, site, and histologic type along with histologic grading were present in all pathology reports. It is noted that neoadjuvant therapy and mesorectal excision is the standard of care for rectal cancer below the anterior peritoneal reflection, hence the anatomic site of the tumor should be known. Histologic variants like signet-ring cell and poorly differentiated neuroendocrine type of colorectal carcinoma are linked with adverse prognostic significance regardless of the tumor stage [11,12]. A high level of microsatellite instability (MSI-H) is strongly associated with medullary histologic type; therefore this histologic type has a better prognosis. It can occur either sporadically or in association with Lynch syndrome [13]. Lymphovascular invasion and lymph node metastasis are strongly associated with micropapillary histologic variants [13]. Various studies revealed that a poorly differentiated grade of the tumor is associated with poor outcomes [14]. In our study, only 25% of reports indicated treatment effects. Studies revealed that neoadjuvant CCRT (chemotherapy plus radiotherapy) can cause a remarkable reduction of tumor size in rectal cancer [15]. A resected specimen that shows complete eradication of the tumor (R0 resection) is associated with a good prognosis [16]. Patients with R2 resection (macroscopic diseases) have the worst prognosis than patients with R1 resection (microscopic disease) [17]. Other less reported parameters in our study include TNM descriptors and tumor deposits. It is obvious from the results that 39.2% of the reports commented about tumor deposits. The presence of tumor deposits in surgically resected specimen indicates an adverse prognosis [18,19]. Even in the absence of regional lymph node involvement, the tumor will be indicated as N1c due to the presence of tumor deposits. Nancy G Chan et al., in 2008 conducted a study to assess the quality of surgical pathology reports. This study concludes that few important parameters required for staging and prognostic purposes were under-reported. But after few changes in the reporting format, significant improvement was noticed [20]. In our study, margin details of resected tumors were present in 75% of the reports. Positive radial margin increases the risk of local recurrence in rectal cancer [21]. Various studies revealed that lymphovascular invasion is an indicator of poor outcome and risk of lymph node metastasis [22]. Our study revealed that details of lymphovascular invasion were present in 93% of the reports. Perineural invasion is a poor prognostic marker [23] and our study showed that details of perineural invasion were present in 81.6% of the reports. Perforation of the intestine proximal to an obstructing tumor is a rare complication of colorectal cancer; however, it increases the risk of mortality secondary to sepsis [24]. According to our study, details of tumor perforation were present in only 51.6% of the pathology reports. Total mesorectal excision (TME) results in improved overall survival in addition to the reduction of disease recurrence [25]. However, in this study, 53.5% of reports have documented the evaluation of mesorectum. In the United States, surgical resection of a minimum of 12 lymph nodes is enough according to the National Quality Forum lists (see http://www.facs.org/cancer/qualitymeasures.html). The possibility of diagnosing stage IV disease increases with the number of lymph nodes examined; hence excision of 12 lymph nodes should be considered the minimum target, but all possible lymph nodes should be excised and examined [26]. Our study showed that 85% of the reports described regional lymph node status. For accurate TNM staging, tumor size and lymph node status should be correctly known. Information provided by the surgical pathology reports will help in deciding the adjuvant treatment plan of patients. Pathological staging was present in 91.5% of the reports. Please note that root words "m", "y", and "r" are TNM descriptors. The "m” suffix indicates multiple primary tumors at a single site. The prefix “y” indicates pathological staging after neoadjuvant therapy; and, the prefix “r” indicates a recurrent tumor. According to our results, only 35.9% of the reports documented TNM descriptors.

The development and adoption of a standardized checklist are simple but effective means to assure report adequacy and consistent communication between medical oncologists and pathologists. In addition to accompanying criteria for its proper use a practice guideline for the surgical pathology examination of all resected malignant tumors. In few cases, the pathologist also accounts for inadequate reporting of multiple parameters; therefore, a well-defined checklist will be given to ensure that all parameters are reported accordingly. Bettina Casati and Roger Bjugn (2012) study compared the use of text pathology reports with electronic pathology reports of colorectal cancer to evaluate the presence of all necessary parameters (elements). All histopathology reports before implementation were evaluated concerning the presence of key elements. Similarly, all histopathology reports were evaluated after the implementation of new electronic templates with the presence of all important key elements. Results showed that electronic template reporting had a significant and sustainable long-term, positive effect on the quality of histopathology reports [27]. Leila et al. (2013) conducted a study in which they investigated the prognostic value of total lymph nodes identified and the ratio of lymph nodes in patients who underwent curative resection of colorectal cancer. This study showed that a lower total of lymph nodes identified and a higher ratio of lymph nodes was associated with poor outcomes. In addition, tumor stage was a more important prognostic factor than node stage in patients with inadequate lymph nodes evaluation [28].

The adequacy of reporting varies among different hospitals. Many clinicians have relied upon the College of American Pathologists (CAP) guidelines; however, few clinicians felt the need for minor editing to increase the adequacy of pathology reports. Scott A Renshaw et al. (2014) study concluded that the quality of surgical pathology reports was improved by simplifying and highlighting the necessary elements (parameters) in the format form of pathology report [29]. The limitation of our study is the fewer number of reports in addition to the medical record-based study.

## Conclusions

The results of our study revealed that the majority of surgical pathology reports of colorectal cancer were not up to the standard of CAP guidelines. Multiple salient parameters (elements) required to decide future management plans were missing from the pathology reports. This study emphasizes the need for a standardized checklist for adequate pathology reporting according to international standards. Furthermore, multidisciplinary tumor board meetings will be helpful to improve pathologist's awareness and understanding of the importance of adequate quality pathology reporting in all cancer patients.

## References

[1] (2007). Food, Nutrition, Physical Activity, and the Prevention of Cancer: a Global Perspective. https://discovery.ucl.ac.uk/4841/1/4841.pdf.

[2] Janout V, Kollárová H (2001). Epidemiology of colorectal cancer. Biomed Pap Med Fac Univ Palacku Olomouc Czech Repub.

[3] Ries L AG, Melbert D, Krapcho M (2008). SEER cancer statistics review, 1975-2005. Bethesda, MD.

[4] O'Connell JB, Maggard MA, Livingston EH, Yo CK (2004). Colorectal cancer in the young. Am J Surg.

[5] Bhurgri Y, Khan T, Kayani N (2011). Incidence and current trends of colorectal malignancies in an unscreened, low risk Pakistan population. Asian Pac J Cancer Prev.

[6] Minsky BD, Mies C, Recht A, Rich TA, Chaffey JT (1988). Resectable adenocarcinoma of the rectosigmoid and rectum. I. Patterns of failure and survival. Cancer.

[7] Hermanek P, Sobin LH. (1995). Colorectal carcinoma. Prognostic factors in cancer..

[8] Slevin ML (1996). Adjuvant treatment for colorectal cancer. BMJ.

[9] Krook JE, Moertel CG, Gunderson LL (1991). Effective surgical adjuvant therapy for high-risk rectal carcinoma. N Engl J Med.

[10] Compton CC (2003). Colorectal carcinoma: diagnostic, prognostic, and molecular features. Mod Pathol.

[11] Kang H, O'Connell JB, Maggard MA, Sack J, Ko CY (2005). A 10-year outcomes evaluation of mucinous and signet-ring cell carcinoma of the colon and rectum. Dis Colon Rectum.

[12] Bernick PE, Klimstra DS, Shia J (2004). Neuroendocrine carcinomas of the colon and rectum. Dis Colon Rectum.

[13] Pyo JS, Sohn JH, Kang G (2016). Medullary carcinoma in the colorectum: a systematic review and meta-analysis. Hum Pathol.

[14] Barresi V, Reggiani Bonetti L, Ieni A, Domati F, Tuccari G (2015). Prognostic significance of grading based on the counting of poorly differentiated clusters in colorectal mucinous adenocarcinoma. Hum Pathol.

[15] Ruo L, Tickoo S, Klimstra DS (2002). Long-term prognostic significance of extent of rectal cancer response to preoperative radiation and chemotherapy. Ann Surg.

[16] Gavioli M, Luppi G, Losi L (2005). Incidence and clinical impact of sterilized disease and minimal residual disease after preoperative radiochemotherapy for rectal cancer. Dis Colon Rectum.

[17] Ryan R, Gibbons D, Hyland JM (2005). Pathological response following long-course neoadjuvant chemoradiotherapy for locally advanced rectal cancer. Histopathology.

[18] Lo DS, Pollett A, Siu LL, Gallinger S, Burkes RL (2008). Prognostic significance of mesenteric tumor nodules in patients with stage III colorectal cancer. Cancer.

[19] Puppa G, Maisonneuve P, Sonzogni A (2007). Pathological assessment of pericolonic tumor deposits in advanced colonic carcinoma: relevance to prognosis and tumor staging. Mod Pathol.

[20] Chan NG, Duggal A, Weir MM, Driman DK (2008). Pathological reporting of colorectal cancer specimens: a retrospective survey in an academic Canadian pathology department. Can J Surg.

[21] Nagtegaal ID, Marijnen CA, Kranenbarg EK, van de Velde CJ, van Krieken JH (2002). Circumferential margin involvement is still an important predictor of local recurrence in rectal carcinoma: not one millimeter but two millimeters is the limit. Am J Surg Pathol.

[22] Lim SB, Yu CS, Jang SJ, Kim TW, Kim JH, Kim JC (2010). Prognostic significance of lymphovascular invasion in sporadic colorectal cancer. Dis Colon Rectum.

[23] Liebig C, Ayala G, Wilks J (2009). Perineural invasion is an independent predictor of outcome in colorectal cancer. J Clin Oncol.

[24] Anwar MA, D'Souza F, Coulter R, Memon B, Khan IM, Memon MA (2006). Outcome of acutely perforated colorectal cancers: experience of a single district general hospital. Surg Oncol.

[25] Arbman G, Nilsson E, Hallböök O, Sjödahl R (1996). Local recurrence following total mesorectal excision for rectal cancer. Br J Surg.

[26] Cserni G, Vinh-Hung V, Burzykowski T (2002). Is there a minimum number of lymph nodes that should be histologically assessed for a reliable nodal staging of T3N0M0 colorectal carcinomas?. J Surg Oncol.

[27] Casati B, Bjugn R (2012). Structured electronic template for histopathology reporting on colorectal carcinoma resections: five-year follow-up shows sustainable long-term quality improvement. Arch Pathol Lab Med.

[28] Ghahramani L, Razzaghi S, Mohammadianpanah M, Pourahmad S (2013). Adequacy of lymph node staging in colorectal cancer: analysis of 250 patients and analytical literature review. Ann Colorectal Res.

[29] Renshaw SA, Mena-Allauca M, Touriz M, Renshaw A, Gould EW (2014). The impact of template format on the completeness of surgical pathology reports. Arch Pathol Lab Med.

